# MLTSS AND AGING IN PLACE: HOW PERSONAL CARE SERVICES HELP AVOID NURSING FACILITY ADMISSIONS OF OLDER ADULTS

**DOI:** 10.1093/geroni/igz038.2703

**Published:** 2019-11-08

**Authors:** Lycia Tramujas Vasconcellos Neumann, Patricia I Documet

**Affiliations:** University of Pittsburgh, Pittsburgh, Pennsylvania, United States

## Abstract

Older adults value community living and prefer to “age in place”, which increased the need for long-term care to be provided at home. Recently, a capitated option for Medicaid beneficiaries, managed long-term services and supports (MLTSS), has become popular. This evaluation research study investigated 1) the effectiveness of attendant care services to avoid long-term institutionalization of older adults when provided as part of an MLTSS program, and 2) the effect of the type of attendant care services on long-term institutionalization. Using enrollment and claims data of 491 community-dwelling older adults enrolled in an MLTSS program for at least six months, multivariate regression models analyzed the association between long-stay nursing facility (LSNF) admissions and the use of attendant care services. Findings confirmed the hypothesis that those receiving attendant care services are less likely to have LSNF admissions and that as the dosage increases, the odds of LSNF decreased. The type of attendant care services also influenced the results. Participants who used only self-directed AC services were 93% less likely to have an LSNF admission than those receiving no AC services, and 23.5% less likely to have this outcome than those who only received agency attendant care services. In addition, the use of other home and community-based services are also significantly associated with LSNF admissions. These findings have important research and practical implications at the program and policy level, as MLTSS programs spread across the country and aim to “rebalance” the LTSS system.

